# Five-Year Impact of Different Multi-Year Mass Drug Administration Strategies on Childhood *Schistosoma mansoni*–Associated Morbidity: A Combined Analysis from the Schistosomiasis Consortium for Operational Research and Evaluation Cohort Studies in the Lake Victoria Regions of Kenya and Tanzania

**DOI:** 10.4269/ajtmh.19-0273

**Published:** 2019-08-12

**Authors:** Ye Shen, Ryan E. Wiegand, Annette Olsen, Charles H. King, Nupur Kittur, Sue Binder, Feng Zhang, Christopher C. Whalen, William Evan Secor, Susan P. Montgomery, Pauline N. M. Mwinzi, Pascal Magnussen, Safari Kinung’hi, Carl H. Campbell, Daniel G. Colley

**Affiliations:** 1 Department of Epidemiology & Biostatistics, University of Georgia, Athens, Georgia;; 2 Parasitic Diseases Branch, Division of Parasitic Diseases and Malaria, Centers for Disease Control and Prevention, Atlanta, Georgia;; 3 Swiss Tropical and Public Health Institute, Basel, Switzerland;; 4 University of Basel, Basel, Switzerland;; 5 Section for Parasitology and Aquatic Pathobiology, Faculty of Health and Medical Sciences, University of Copenhagen, Copenhagen, Denmark;; 6 Center for Global Health and Diseases, Case Western Reserve University, Cleveland, Ohio;; 7 Schistosomiasis Consortium for Operational Research and Evaluation, Center for Tropical and Emerging Global Diseases, University of Georgia, Athens, Georgia;; 8 Centre for Global Health Research, Kenya Medical Research Institute, Kisumu, Kenya;; 9 Centre for Medical Parasitology, Faculty of Health and Medical Sciences, University of Copenhagen, Copenhagen, Denmark;; 10 Mwanza Research Centre, National Institute for Medical Research, Mwanza, Tanzania;; 11 Department of Microbiology, University of Georgia, Athens, Georgia

## Abstract

The WHO recommends mass treatment with praziquantel as the primary approach for *Schistosoma mansoni*–related morbidity control in endemic populations. The Schistosomiasis Consortium for Operational Research and Evaluation implemented multi-country, cluster-randomized trials to compare effectiveness of community-wide and school-based treatment (SBT) regimens on prevalence and intensity of schistosomiasis. To assess the impact of two different treatment schedules on *S. mansoni*–associated morbidity in children, cohort studies were nested within the randomized trials conducted in villages in Kenya and Tanzania having baseline prevalence ≥ 25%. Children aged 7–8 years were enrolled at baseline and followed to ages 11–12 years. Infection intensity and odds of infection were reduced both in villages receiving four years of annual community-wide treatment (CWT) and those who received biennial SBT over 4 years. These regimens were also associated with reduced odds of undernutrition and reduced odds of portal vein dilation at follow-up. However, neither hemoglobin levels nor the prevalence of the rare abnormal pattern C liver scores on ultrasound improved. For the combined cohorts, growth stunting worsened in the areas receiving biennial SBT, and maximal oxygen uptake as estimated by fitness testing scores declined under both regimens. After adjusting for imbalance in starting prevalence between study arms, children in villages receiving annual CWT had significantly greater decreases in infection prevalence and intensity than those villages receiving biennial SBT. Although health-related quality-of-life scores improved in both study arms, children in the CWT villages gained significantly more. We conclude that programs using annual CWT are likely to achieve better overall *S. mansoni* morbidity control than those implementing only biennial SBT.

## INTRODUCTION

Schistosomiasis remains a major public health problem in much of Africa. The clinical consequences of *Schistosoma mansoni* infections result from tissue damage and blood loss caused by schistosome eggs trapped in host tissues.^1^ Chronic immunologic reactions to the eggs cause granuloma formation in the intestines, liver, and spleen and can progress to cause hepatic and splenic enlargement, periportal fibrosis, portal hypertension, and esophageal varices.^2^ Other impacts of infection, particularly in children, include anemia, malnutrition, impaired growth, impaired cognitive development, and generalized body weakness.^1,3^

Current WHO guidelines call for mass treatment with praziquantel in endemic communities to achieve morbidity control.^4–6^ However, questions remain about optimal programmatic implementation of mass drug administration (MDA).

The overall goal of the Schistosomiasis Consortium for Operational Research and Evaluation (SCORE) project (https://score.uga.edu/) is to provide an evidence base for programmatic decision-making related to control and elimination of schistosomiasis.^7^ Among the studies in the SCORE portfolio were multi-arm, multi-year, randomized intervention trials that assessed changes in prevalence and intensity of schistosomiasis in children aged 9–12 years in villages receiving MDA using different strategies over a 4-year intervention period.^7^ The results from the randomized trials in Kenya and Tanzania have been reported elsewhere.^8,9^

**Figure 1.**
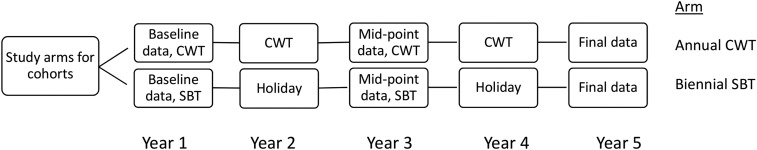
Flow diagram of the cohort study. In the upper arm, children lived in communities randomized to receive annual community-wide mass drug administration treatment (CWT). The children in the lower study arm lived in communities randomized to receive only biennial (every other year) school-based treatment (SBT), with drug “holidays” in Year 2 and in Year 4. Final assessment for both groups was performed in Year 5 of the study.

These longitudinal studies also provided an opportunity to explore the impact of MDA on schistosomiasis-associated morbidity in children.^10^ Therefore, SCORE nested cohort studies of morbidity within intervention trials in Kenya and Tanzania that were occurring in villages that had prevalence of ≥ 25% *S. mansoni* infection during village eligibility testing.^10^ The treatment regimens in the two study arms that included cohorts were either 1) 4 years of annual community-wide treatment (CWT) or 2) every-other-year school-based treatment (SBT) (Figure 1). In the present analysis, we combined the data from the *S. mansoni* cohorts in Kenya and Tanzania to assess and compare the impact of the two regimens on a range of infection-associated morbidity markers. Although results of analysis of the Kenya cohort^11^ and the baseline data from the Tanzania cohort^12^ have been previously published, the present secondary analysis, combining the Kenya and Tanzania data, increases the statistical power to detect significant differences between CWT and SBT regimens. The current study describes changes in anthropometric growth indices, hemoglobin (Hb) levels, measures of physical fitness, and quality of life, as well as the liver abnormalities and portal vein findings among study cohort children (aged 7–8 years at baseline) at the end of their 4-year study participation.^10^ Our hypothesis was that annual CWT with praziquantel could provide incremental health benefits in terms of reductions in observed morbidity when compared with the effects of every-other-year treatment administered in a school-based program.^10^

## MATERIALS AND METHODS

### Ethics statement on subject recruitment.

Approval for the SCORE in Kenya and Tanzania intervention studies (the “gaining control” studies) and their related cohort studies was obtained from the Institutional Review Boards at the Kenya Medical Research Institute (Nairobi, Kenya), the Centers for Disease Control and Prevention (Georgia), and the Medical Research Coordination Committee of the National Institute for Medical Research (Tanzania). Trials Registration numbers are ISRCT 16755535 (Kenya) and ISRCT 95819193 (Tanzania).^7^ Only children who assented to participate and had written informed consent from parents or their legally authorized representatives were eligible for inclusion. Before examination and sample collection, the reason for the survey and the procedures for sample collection were explained to the children and the adult population in the communities, as well as local leaders, school administrators, teachers, and health and education personnel.

### Study area and population.

The results reported in this article are a secondary analysis of data combined from parallel cohort studies that took place in the Nyanza region (Siaya, Kisumu, and Homa Bay counties) of Kenya^13^ and the Mwanza region (Misungwi and Sengerema districts) of Tanzania,^9^ both of which have high prevalence of *S. mansoni*. All study villages were located on or near the Lake Victoria shoreline.

The cohort study design is described in detail in earlier articles.^10,12,14^ Briefly, the cohort studies were nested in larger intervention trials on gaining control of schistosomiasis mansoni in moderate- to high-risk communities. In these parent SCORE intervention trials, 150 villages per country were randomized to one of six treatment arms and given MDA using different approaches (CWT versus SBT) at different frequencies (either two or four treatments) over a 4-year period.^7^ Villages for inclusion in the nested cohort studies reported in this article came from the 25 villages in the treatment arm with annual CWT (the most intense treatment arm) and the 25 villages with every-other-year SBT (a less intense treatment arm) (see Figure 1 for flow diagram).^10^ To achieve the target of enrolling 800 7–8-year-old children in each country, Tanzania included three of the 25 villages in the annual CWT arm and four of the 25 in the biennial SBT arm, whereas Kenya included six villages from each arm.^10^ Parasitologic and morbidity parameters were measured at baseline and in Year 5, and although some measures were made in Year 3, these were not uniform between sites and are not included in the present analysis. In years when villages were scheduled to receive treatment, the protocol called for efforts to treat all village school-age children, whether in school or not, and irrespective of their egg-positive or egg-negative status. In CWT villages, schools were used as a supplemental venue to locate children not found at home. In SBT villages, community mobilization teams were used to encourage parents to have their children who were not attending school come to receive treatment at the school on a subsequent day. During praziquantel drug holiday years, children having symptoms could seek evaluation and treatment at health facilities, which sometimes were able to provide individual praziquantel treatment. However, few, if any, children received treatment during drug holiday years. There was no untreated comparison group. Only those children who participated in both Year 1 and Year 5 are included in the analyses presented in this article.

### Data on parasitology and morbidity.

The methods for the collection of cohort data are described in detail elsewhere^12,14^ but are briefly presented in the following paragraphs.

#### Stool sample collection and examination.

Participants provided stool specimens on each of three consecutive days. Duplicate Kato–Katz thick smears were made with a 41.7-mg template^15^ from each specimen and examined for *S. mansoni* eggs by trained microscopists.

#### Blood collection and Hb assessment.

A 5-mL tube was used to collect 2–3 mL of venous blood sample (Kenya) or a finger-prick blood sample (Tanzania) was collected from each individual, and the Hb level was measured using a portable HemoCue photometer (HemoCue, Inc., Ängelholm, Sweden). The Hb level was reported in gm/dL, and final values used in analysis were adjusted for altitude (ca. 1,000 m) by subtracting 2 gm/dL from the raw values for both study sites.^16^ Anemia was defined as Hb values < 11.5 gm/dL for children younger than 12 years and Hb < 12 gm/dL for children of 12 years and older but younger than 15 years, according to the WHO guidelines.^17^

#### Anthropometric measurements.

Height was measured for barefoot children using a wooden stadiometer. The child looked straight ahead while standing on the base of the stadiometer with their heels, buttocks, shoulder blades, and back of the head touching the vertical backboard. Once the child was correctly positioned, the stadiometer head plate was lowered and the height measured in centimeters to one decimal place. Weight was measured on a digital scale in kilograms to one decimal place for barefoot children after excess clothing was removed. Height and weight were measured twice by the same examiner and the mean value was recorded. *Z*-scores were calculated using WHO AnthroPlus software (available at https://www.who.int/growthref/tools/en/) based on the WHO growth reference data tables for 5–19-year-old children.^18^ In Tanzania, the exact birthdays of some children (and hence, their exact age in days) were not known. For such cases, the midpoint of the *Z*-score limits was used, for example, for children reported to be 7 years old, the *Z*-score for children aged 7 years and 6 months was used. Wasting was defined as a body mass index-for-age *Z*-score of ≤ −2 and stunting as a height-for-age *Z*-score ≤ −2.

#### Physical fitness.

Physical fitness was assessed using the 20-m shuttle run fitness test (20mSRT) as described by Bustinduy et al.^19^ In brief, during the test, children run continuously between two lines that are 20 m apart.^20,21^ A run from one line to the other is considered a shuttle. There are 21 levels in the test, and the higher the level, the greater the number of shuttles and faster the pace required to complete it. The running field was prepared in the school compound and runners were laterally separated by at least 1 m. Recorders were placed at each end of the field, and every recorder was responsible for monitoring three to five children. The recorder noted the level at which the test subject stopped and how many shuttles the child had completed within that level. These numbers were converted to a maximal oxygen uptake, the VO_2_ max (maximal oxygen uptake as estimated by fitness testing), in mL/kg/minute, as previously described.^22,23^

#### Quality of life.

Quality of life was assessed using the Pediatric Quality of Life Inventory instrument (PedsQL) for children,^24,25^ which, although developed in the Unites States, has been validated for health-related quality-of-life assessment in the East African setting.^12,14,26,27^ Kenya used a 23-question version of the PedsQL survey,^14^ and Tanzania used a 16-question version, but discarded the last question because of irrelevance to the local setting.^10,12^ The PedsQL questionnaire is divided into four parts, with three to six questions in each section. The four parts describe four dimensions of functioning: 1) problems with physical activity (physical), 2) problems with feelings (emotional), 3) problems with getting along with others (social), and 4) problems with keeping up in school (school). The answers are scored on a Likert-like scale from 0 to 4, where 0 is never, 1 is almost never, 2 is sometimes, 3 is often, and 4 is almost always. Responses are transformed to scores that range from 0 to 100, with higher scores indicating a better perceived quality of life.

#### Abdominal ultrasonography.

Abdominal ultrasound was performed using portable ultrasound machines (Aloka Sonocamera SSD-500 with a 3.5 MHz curvilinear probe, Hitachi Aloka Medical America, Wallingford, CT) in both Kenya and Tanzania. The examinations were performed according to the WHO’s Niamey protocol for imaging schistosomiasis^28^ by senior sonographers with extensive experience in the field of ultrasonography of *S*. *mansoni*–infected individuals. Children were examined while lying on their backs on an examination table with their legs extended. Measurements included length of the left liver lobe (mm), spleen length (mm), portal branch thickness, and portal-vein diameter. The liver image was scored as one of six patterns, A–F, as described in the WHO protocol.^28^ Image patterns A and B are considered normal or nonspecific. Image patterns C and D are considered characteristic of mild and moderate *S. mansoni* infection–related fibrosis, respectively, whereas liver patterns E and F indicate advanced infection-related liver fibrosis. Increased portal vein diameter was defined as 2 SD above standard reference measurements developed from healthy, uninfected children of corresponding height in other endemic countries.^28,29^

### Statistical analysis.

Subjects were considered positive for infection if at least one egg was found on any of the Kato–Katz slides prepared from their stool specimens. The mean egg count for the six slides was calculated and multiplied by 24 to estimate the child’s infection intensity in eggs per gram of stool (epg). In Kenya, egg counts were truncated at 42 eggs per slide, indicating a heavy infection having > 1,000 epg. In cases where specimens were missing, the calculation was performed using data from the available slides for each affected child. Consistent with the WHO guidelines,^4^ infected individuals with < 100 epg were considered to have light infections, those with 100–399 epg to have moderate intensity infections, and individuals with ≥ 400 epg to have severeinfections. For the present analysis, group-wise infection intensity is reported in two different ways: 1) as the arithmetic mean of epg for all tested persons, including those with epg = zero (mean intensity for the entire cohort) and 2) as the mean of epg only for those children found to be egg positive (mean intensity among those infected). Absolute change in prevalence from Year 1 to Year 5 was calculated as (prevalence in Year 5 minus prevalence in Year 1). For example, a 20% decrease in prevalence in a location having a starting prevalence of 40% means a decline to 20% prevalence, whereas in a location with a starting prevalence of 80%, 20% prevalence reduction would result in a prevalence of 60%. Relative percent change in prevalence from Year 1 to Year 5 was calculated as ([prevalence in Year 5 minus prevalence in Year 1/prevalence Year 1] × 100). This would determine the relative percent change in prevalence, regardless of starting prevalence. Using this method, the starting prevalence would influence the level of decline, such that going from 80% to 60% would be a 25% drop in prevalence, whereas going from 40% to 20% would be classified as a 50% decline in prevalence.

Summary statistics were calculated to compare the characteristics of the combined cohort in terms of demography, infection status, and morbidity markers and to compare those who had only Year 1 data (i.e., those lost to follow-up) with those who remained in the cohort in Year 5. Following the SCORE project’s a priori statistical analysis plan, linear or generalized linear mixed effect models adjusting for village-level clustering effects, and, where appropriate, age and gender were used to obtain odds ratios (for binary outcomes) and group-wise differences (for continuous outcomes) for comparisons between study arms. Even though all villages in the Kenyan and Tanzanian locations were selected based on having a baseline school age prevalence of infection of ≥ 25%, because of the village-level cluster randomization design, there were resultant imbalances in baseline infection factors (average prevalence and intensity) between study arms. As a result, our ability to detect differences by treatment arm at Year 5 was diminished. To compensate for these starting imbalances and to better detect differences between the two arms from Year 1 to Year 5, we also studied the interaction effects of survey year with study arm on relative changes in results for infection, infection intensity, and morbidity markers, although adjusting for gender. For outcome variables missing in 10% or more children from the entire cohort, analyses initially conducted for complete cases were later repeated with missing data imputed using multiple imputation procedures.^10,30^ Results obtained from the multiply-imputed datasets were compared with those generated from the original dataset. Statistical analyses were performed using SAS version 9.4 (SAS Institute Inc., Cary, NC). An α = 0.05 level was used for significance of all statistical tests and for the confidence interval calculations.

## RESULTS

### Combined cohort characteristics.

**Figure 2.**
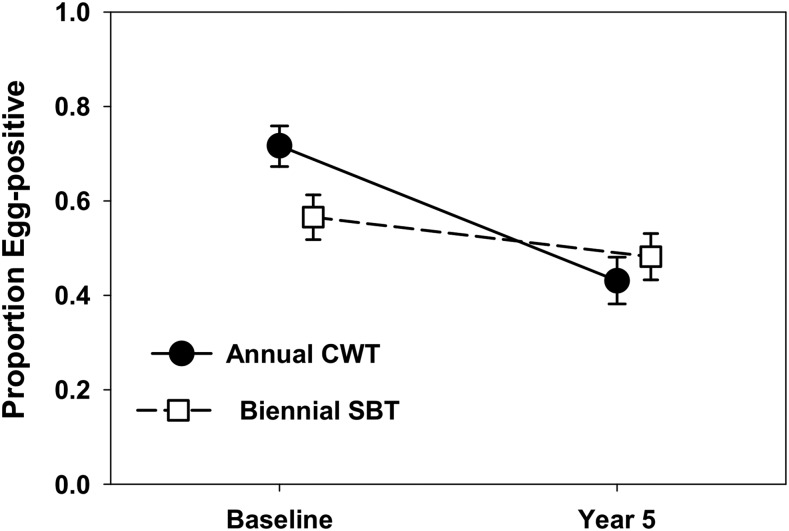
Comparison of Year 1 and Year 5 *Schistosoma mansoni* prevalence for participating children in each of the combined cohort study arms (annual community-wide treatment (CWT) vs. biennial school-based treatment [SBT]). Dark circles indicate baseline and Year 5 prevalence values for the annual CWT arm. Open squares indicate corresponding prevalence values for the children in the biennial SBT arm. Error bars indicate 95% CI.

**Figure 3.**
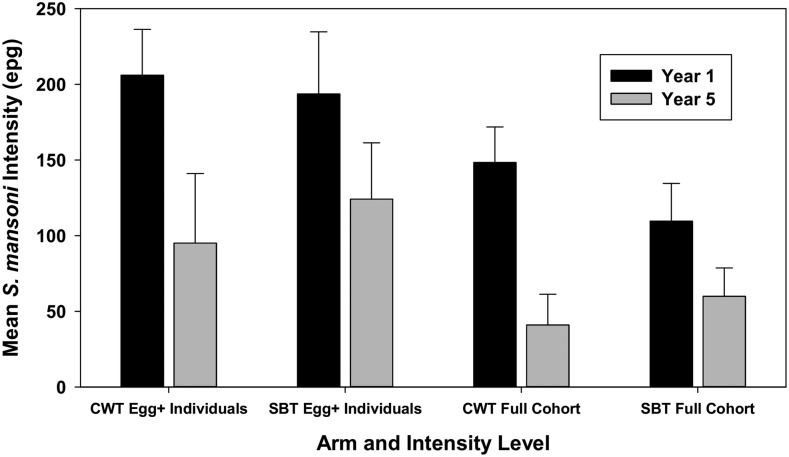
Comparison of Year 1 and Year 5 *Schistosoma mansoni* infection intensities by the cohort study arm. Shown here are Year 1 baseline intensity values (dark bars) and 95% CI for participating children in the two study arms receiving either annual community-wide treatment (CWT) or biennial school-based treatment (SBT), calculated either as arithmetic mean intensity for egg-positive children (individual-level intensity, left side), or as mean intensity for all children, including those with zero egg counts (cohort-level intensity, right side). Corresponding values for participating children in Year 5, after MDA, are shown by the light bars.

Table 1 describes the characteristics of participants in Year 1 at baseline, and in Year 5 after 4 years of intervention. Of 1,374 individuals who were enrolled at baseline in the combined cohort, 891 (64.5%) participated in Year 5 data collection. In aggregate, girls comprised 55% of participants in the annual CWT arm and 50% of participants in the biennial SBT arm at both baseline and Year 5 (difference not significant [NS], *P* = 0.118). At baseline (Year 1), 72% of participants in the annual CWT arm and 57% in the biennial SBT arm were infected (Figure 2), and this difference was statistically significant (*P* < 0.0001). Mean intensities for the entire cohort at baseline were 148 epg for children in the annual CWT arm, which is significantly higher than the 110 epg for those in the biennial SBT arm (Figure 3). Baseline individual-level intensity, including only those children who were egg positive, was 206 epg in the annual CWT arm and 194 epg in the biennial SBT arm (difference NS).

**Table 1:** Baseline and Year 5 characteristics of cohort participants (children with data both in Year 1 and Year 5) and baseline characteristics of those lost to follow-up, by arm

	Year 1, cohort children		Year 5, cohort children		Year 1 data for children lost to follow-up in Year 5	
	Annual CWT arm*	Biennial SBT arm*	Annual CWT arm	Biennial SBT arm	Annual CWT arm	Biennial SBT arm
Number of children	450	441	450	441	220	263
Percent female	54.9%	49.7%	54.9%	49.7%	54.1%	50.2%
Age (years) (SD)	7.4 (0.5)	7.6 (0.5)	11.5 (0.5)	11.6 (0.5)	7.5 (0.5)	7.6 (0.5)
No. tested for schistosomiasis	446	438	406	413	217	252
No. infected	320	248	175	199	154	122
Prevalence	72.1%	56.6%	43.1%	48.2%	71%	48.4%
Full cohort arithmetic mean infection intensity (epg)	148.4	109.7	41	59.9	144.9	113.8
Egg-positive children’s mean infection intensity (epg)	206	193.7	95.1	124.2	204.1	235

CWT = community-wide treatment; epg = eggs per gram feces; SBT = school-based treatment.

* After the baseline (Year 1) survey, communities in the annual CWT arm received community-wide praziquantel treatment every year for four years; communities in the biennial SBT arm received SBT every other year.

By Year 5, prevalence had declined to 43% in the annual CWT arm and 48% in the biennial SBT arm (difference NS, Figure 2). Both cohort-level and individual-level infection intensities declined in both arms, with relatively higher egg-reduction rates in the annual CWT arm (Figure 3). Baseline characteristics of lost-to-follow-up subjects were similar to those of children who had remained in the study through Year 5 (Table 1).

The absolute differences in prevalence values between Year 5 and Year 1 in the annual CWT arm and in the biennial SBT arm were 29 (CI_95%_ = 22.6–35.3) and 8.4 (CI_95%_ = 1.8–15.1) percentage points, and the relative changes in prevalence for the two arms were 40.2% and 14.8% reductions, respectively. Absolute differences for full cohort mean intensity for treatment subgroups were 107.4 (CI_95%_ = 76.2–138.6) epg in the annual CWT arm and 49.8 (CI_95%_ = 18.4–81.3) epg in the biennial SBT arm, with corresponding relative reductions of 72.4% and 45.4%. Absolute changes in individual-level intensity of egg-positive children in the two arms were 110.8 (CI_95%_ = 57.7–163.9) epg and 69.5 (CI_95%_ = 13–126) epg, and their relative changes were 53.8% and 35.9%, respectively. In each category, the annual CWT arm subjects had greater declines in these measures. Between Year 1 and Year 5, prevalence of heavy infections declined from 11.1% to 1.7% in the annual CWT arm and from 8.9% to 2.6% in the biennial SBT arm. After adjustment for village-level clustering effects, these between-arm differences in heavy intensity prevalence were not significant (Rao–Scott χ^2^ = 0.116, *P* = 0.73 for between-arm difference in Year 1; χ^2^ = 1.07, *P* = 0.30 for between arm difference in Year 5).

We observed a significant increase in stunting among children in the biennial SBT arm, and significant decreases in wasting prevalence in both arms over the course of the study (Table 2). Hemoglobin levels did not change significantly during the study period. The prevalence of anemia remained roughly stable between Year 1 and Year 5 in the annual CWT arm and declined slightly, but not significantly, in the biennial SBT arm. Maximal oxygen uptake as estimated by fitness testing scores dropped in both arms from Year 1 to Year 5. Among PedsQL outcomes, the total score, as well as scores on the physical, emotional, and school subdomains, increased over time in both study arms. There were significant Year 1 to Year 5 declines in the percentage of children with elevated portal vein diameter in both study arms. Abnormal liver pattern (Pattern C or higher) was rare, and no significant changes were noted for this finding in either arm between Year 1 and Year 5.

**Table 2:** Summary results for morbidity markers, by arm, by year

	Annual CWT arm		Biennial SBT arm	
	Year 1	Year 5	Year 1	Year 5
Body mass index: Average *Z* score (SD)	−1.3 (1.1)	−0.9 (1.1)	−1 (1)	−0.3 (1)
Prevalence of stunting (CI_95%_)	9.9% (7.1, 12.7%)	11.5% (8.4, 14.6%)	5.1% (3, 7.1%)	10.8% (7.9, 13.8%)
Prevalence of wasting (CI_95%_)	24.9% (20.9, 29%)	12.7% (9.5, 15.9%)	13.2% (10, 16.4%)	3.9% (2, 5.7%)
Mean hemoglobin gm/dL (SD)	12 (2.3)	11.7 (1.8)	11.9 (2.1)	12.1 (1.8)
Prevalence of anemia (CI_95%_)	42.6% (37.9, 47.2%)	45.8% (40.8, 50.7%)	42.9% (38.2, 47.6%)	37.9% (33.3, 42.6%)
Mean VO_2_ max score (SD)	49.2 (3.3)	43.5 (6.2)	49.2 (3.4)	44.4 (5.8)
Mean pediatric quality of life inventory scores				
Total score (SD)	83.6 (12.4)	91.9 (11.6)	85 (14.3)	90.7 (11.8)
Physical (SD)	88.9 (14.9)	94.5 (11.2)	88.2 (19.7)	93.2 (15.1)
Emotional (SD)	71.9 (15.1)	89.8 (16.1)	77.1 (16.8)	87.1 (18.2)
Social (SD)	90.4 (16.4)	91.9 (16.4)	91.3 (18.6)	93.1 (14.1)
School (SD)	80 (16.3)	90.7 (13.7)	81.6 (18.6)	89.2 (14)
Prevalence of increased portal vein diameter (CI_95%_)	10.8% (7.9, 13.7%)	1.7% (0.4, 2.9%)	9% (6.3, 11.7%)	1.2% (0.2, 2.2%)
Ultrasound pattern prevalence				
Pattern B (CI_95%_)	16.6% (13, 20.1%)	11.4% (7.5, 13.4%)	8.9% (6.2, 11.6%)	11.7% (7.8, 13.7%)
Pattern C or higher (CI_95%_)	0.7% (0.1, 2%)	0.5% (0, 1.7%)	0.9% (0.3, 2.4%)	0.7% (0.1, 2%)

CWT = community-wide treatment; SBT = school-based treatment; VO_2_ max = maximal oxygen uptake as estimated by fitness testing.

### Comparison of infection and morbidity markers between Year 1 and Year 5.

Table 3 presents prevalence, intensity, and the morbidity markers with statistically significant changes between Year 1 and Year 5 for all children in the combined cohorts, regardless of arm assignment (*N* = 891). All comparisons use Year 1 as the reference group. For the combined groups, overall prevalence declined significantly from Year 1 to Year 5, as did the prevalence of heavy infections and infection intensity. Wasting was significantly less common, but stunting was significantly more common in Year 5 relative to Year 1. In addition, the percentage of children with elevated portal vein diameter values declined significantly from Year 1 to Year 5. Maximal oxygen uptake as estimated by fitness testing max decreased significantly from Year 1 to Year 5. By contrast, all the PedsQL measures increased significantly from Year 1 to Year 5.

**Table 3:** Outcomes with significant changes from Year 1 to Year 5, for both study arms combined

	Odds ratio (reference = Year 1)	CI_95%_	*P*-value
Infection prevalence	0.36	0.28, 0.47	< 0.0001
Prevalence of heavy infections	0.22	0.14, 0.36	< 0.0001
Wasting	0.37	0.27, 0.50	< 0.0001
Stunting	1.56	1.10, 2.21	0.02
Elevated portal vein diameter	0.13	0.07, 0.25	< 0.0001

	Average change	CI_95%_	*P*-value
VO_2_ (mL/kg/min)	−5.5	−5.9, −5.2	< 0.0001
Pediatric quality of life inventory			
Total score	8	7, 9.1	< 0.0001
Physical	6.2	4.8, 7.6	< 0.0001
Emotional	14.8	13.3, 16.3	< 0.0001
Social	2.9	1.5, 4.3	< 0.0001
School	10.1	8.7,11.5	< 0.0001

	Coefficient	CI_95%_	*P*-value
Mean cohort-level infection intensity (epg)	−0.92	−0.93, −0.91	< 0.0001

epg = *S. mansoni* eggs per gram feces; VO_2_ max = maximal oxygen uptake as estimated by fitness testing.

### Age-, and gender-adjusted comparisons between study arms, accounting for village-level intraclass correlation.

In this first-stage modeling analysis of the SCORE project’s a priori statistical analysis plan, no significant differences were observed between the two study arms across the range of measured infection and morbidity markers (Table 4).

**Table 4:** Age- and gender-adjusted CWT-arm participant likelihood of study morbidity at Year 5, using the SBT-arm participants as reference

	Odds ratio (reference = the biennial school-based treatment arm)	CI_95%_	*P*-value
Infection prevalence	0.77	0.19, 3.06	0.70
Wasting	2.73	0.94, 7.90	0.06
Stunting	1.19	0.57, 2.47	0.65
Anemia	1.26	0.51, 3.12	0.62
Increased PVD	1.40	0.25, 7.72	0.70

	Arm difference	CI_95%_	*P*-value
VO_2_ (mL/kg/min)	−0.5	−4.8, 3.7	0.80
Pediatric quality of life inventory			
Total score	0.7	−7.1, 8.5	0.86
Physical	0.7	−5.6, 7	0.82
Emotional	1.8	−9.6, 13.2	0.76
Social	−1.4	−9.3, 6.6	0.73
School	1.4	−6.2, 8.9	0.73

	Coefficient	CI_95%_	*P*-value
Mean cohort-level intensity (epg)	−0.1	−1.9, 1.7	0.92

CWT = community-wide treatment; epg = *S. mansoni* eggs per gram feces; PVD = portal vein diameter; VO_2_ max, maximal oxygen uptake as estimated by fitness testing.

To account for the imbalances in baseline disease status, in a secondary analysis, we next studied the interaction effects of the survey year (Year 5 versus Year 1) and study arm (CWT versus SBT) on relative changes in results for the cohort indicators of infection and morbidity, adjusting for gender, village-level clustering effects, and individual Year 1 starting values (Table 5). By this analysis, prevalence and intensity dropped significantly more by Year 5 in the annual CWT arm, as compared with the SBT arm. Maximal oxygen uptake as estimated by fitness testing max levels declined in both the arms, but decreased significantly more in the annual CWT arm than in the biennial SBT arm. By contrast, changes of total PedsQL score and its emotional and school subdomains were all significantly positive from Year 1 to Year 5 for both the arms, but children in the annual CWT arm gained more. Not shown, changes in prevalence of anemia, wasting, stunting, or increased portal vein diameter were not statistically different between the arms (see Supplemental Table 1).

**Table 5:** Gender-adjusted comparison of arm-specific changes in infection and morbidity outcomes from Year 1 to Year 5, including interaction term added to account for differences in starting values between groups

Outcomes	Predictors	Coefficient	CI_95%_	*P*-value
Infection prevalence	Year 5	−0.4	−0.8, −0.1	0.01
	Annual CWT	0.6	−0.7, 1.9	0.35
	Annual CWT × Year 5	−1.1	−1.5, −0.6	< 0.0001
Intensity (epg)	Year 5	−0.59	−0.61, −0.58	< 0.0001
	Annual CWT	0.7	−1.1, 2.6	0.41
	Annual CWT × Year 5	−0.68	−0.70, −0.65	< 0.0001
VO_2_ max	Year 5	−5	−5.5, −4.5	< 0.0001
	Annual CWT	0.4	−2.6, 3.4	0.81
	Annual CWT × Year 5	−1	−1.7, −0.3	0.004
PedsQL total	Year 5	6.1	4.6, 7.6	< 0.0001
	Annual CWT	−2.6	−8.9, 3.6	0.40
	Annual CWT × Year 5	3.9	1.8, 6	0.0003
PedsQL emotional	Year 5	10.2	8.1, 12.3	< 0.0001
	Annual CWT	−6.6	−13.2, −0.1	0.05
	Annual CWT × Year 5	9.4	6.4, 12.4	< 0.0001
PedsQL school	Year 5	7.7	5.8, 9.7	< 0.0001
	Annual CWT	−2.7	−9.3, 3.9	0.43
	Annual CWT × Year 5	4.9	2.1, 7.7	0.0007

CWT = community-wide treatment; epg = *S. mansoni* eggs per gram feces; PedsQL = pediatric quality of life inventory; VO_2_ max = maximal oxygen uptake as estimated by fitness testing.

### Sensitivity analysis for effects of missing data using multiple imputation.

The potential impact of missing data was investigated, and the result from the imputed datasets did not differ significantly.

## DISCUSSION

This combined cohort analysis of morbidity outcomes in Kenya and Tanzania^10,12,14^ demonstrates that regular treatment of schoolchildren is associated with reductions in both *S. mansoni* infection prevalence and mean infection intensity. For schoolchildren who were followed to 11–12 years of age, participation in either annual CWT or biennial SBT programs was also associated with reductions in the prevalence of wasting and portal vein dilation. Health-related quality-of-life scores showed improvement in both treatment groups. These findings suggest cumulative benefits from regular preventive treatment (whether CWT or SBT) can be obtained in *S. mansoni*–endemic communities that are similar to those included in this study. Reduction in portal vein dilation, the study pathology most closely tied to adverse outcomes from intestinal schistosomiasis, is of particular importance. In the absence of an untreated concurrent control group, we cannot definitively ascribe these benefits to treatment intervention. However, prolonged nontreatment was considered to be an unethical choice at the time of study design.^7^ It is thus possible that unmeasured confounding factors such as food insecurity and intercurrent infections or reinfections may have influenced the observed study outcomes.

Because the overall research trial used cluster-based random assignment of villages to treatment regimens, there were differences in baseline *S. mansoni* prevalence and infection intensity between the two arms in our analysis, and this initially obscured the significance of between-arm differences in outcomes, that is, between children in villages receiving annual CWT and those receiving biennial SBT. After adjusting for the higher starting prevalence and intensity, it was noted that annual CWT led to significantly greater decreases in infection prevalence and intensity than did biennial SBT. In addition, children in annual CWT villages gained significantly more in emotional well-being and school satisfaction.

The reasons for persistent morbidity despite MDA are likely to be multifactorial. Location-related variations in reinfection risk, in growth indices, in anemia, and in fitness have been previously demonstrated between the Kenyan and Tanzanian areas included in this study. There were also significant differences in sanitation knowledge and practices and in intake of high-quality protein foods.^31^ Unfortunately, as children grow up in resource-limited areas, cumulative growth deficits are a common finding,^32,33^ and our combined studies’ outcomes are in accord with that frequent observation.

Twenty-meter SRT–based VO_2_ scores have been shown to decline as children age into adolescence, with girls more affected than boys.^19,21^ The higher prevalence of stunting and wasting in the annual CWT arm, both at baseline and in Year 5, may have contributed to the observed between-arm difference in VO_2_ max results, which are affected both by stature and muscle mass. Although socioeconomic status can affect PedsQL scores,^24^ the average scoring does not appear to change as a function of age, either for healthy or chronically ill children and adolescents.^24^ The improvements reported in our cohort analysis are thus unlikely to reflect an age effect and may more likely reflect the impact of continued participation in preventive treatment of chronic schistosomiasis.^5^

There are several limitations to our study. Our study populations live in areas highly endemic for malaria and soil-transmitted helminths, which likely had an impact on anemia outcomes through blood loss, anemia of chronic inflammation,^34^ and intermittent episodes of symptomatic malaria associated with hemolysis.^14,31^ Although both the Kenyan and Tanzanian sites were tested for the presence of coinfection with malaria, the sensitivity of the techniques they used (blood smear versus rapid diagnostic tests) and timing of their testing (Y5 versus Y3) were sufficiently different so that we were unable to estimate the impact of malarial infection across the combined cohort. Perhaps, the most critical study limitation was the parent studies’ village-level cluster randomization design, which provided only a limited number of evaluation units (i.e., villages) available for analysis.^10^ Village-to-village variation in response to MDA schedules proved to be much higher than anticipated in the initial design of the SCORE studies,^11,35^ which likely obscured the differences in MDA impact by the study arm.^9,11^ Therefore, a relative strength of our analysis is the inclusion of a somewhat larger number of study locations, with extended analysis to adjust for differing baseline prevalence between the two study arms. Measurement of infection status only in Year 1 and in Year 5 may not have adequately captured cumulative parasite exposure. A child who tested negative at baseline and in Year 5 could still have spent several years repeatedly infected with *S. mansoni* between the beginning and the end of the study, despite periodic MDA treatments and could have accrued significant residual morbidity without evidence of active infection in Year 5.

The current WHO strategy for reduction in schistosomiasis morbidity is based on achieving reductions in the community-level prevalence of heavy infections among school-age children.^6^ Few children in our cohorts had heavy infections by the WHO definition (≥ 400 epg in feces), so we could not assess whether reduction in intensity changed individual morbidity risk. Nevertheless, even though most of the combined cohort children were not heavily infected, in aggregate, they appeared to benefit significantly from their community’s participation in MDA. It is not clear, however, whether these improvements will remain in effect if MDA is stopped and exposure to infection continues.

Larger studies are currently being designed to better define the community-level infection prevalence and intensity levels below which morbidity associated with schistosomiasis cannot be detected. These studies will more fully measure the spectrum of schistosomiasis-related morbidities, and control for cofactors that contribute to their occurrence. Such additional data should be extremely helpful as the WHO refines its global programmatic guidelines for control of *Schistosoma* infection–related morbidity.

## Supplemental table

Supplemental materials
