# Supplementary material for: Five-Year Impact of Different Multi-Year Mass Drug Administration Strategies on Childhood Schistosoma mansoni–Associated Morbidity: A Combined Analysis from the Schistosomiasis Consortium for Operational Research and Evaluation Cohort Studies in the Lake Victoria Regions of Kenya and Tanzania
Source: Am J Trop Med Hyg. 2019 Aug 12;101(6):1336–44. doi: 10.4269/ajtmh.19-0273 (PMC6896894; doi:10.4269/ajtmh.19-0273)
Supplement: Supplementary file 1 [file tpmd190273.SD1.docx]

**Supplemental Table S1. Non-significant differences in arm-specific changes from Year 1 to Year 5 when the interaction of study arm by survey year is considered^a^**

| **Outcomes** | **Predictors** | **Coefficient** | **CI_95%_** | **P-value** |
| --- | --- | --- | --- | --- |
| **Stunting** | Year 5 | 0.8 | 0.3, 1.4 | 0.002 |
|  | Annual CWT | 0.6 | -0.2, 1.5 | 0.14 |
|  | Annual CWT x Year 5 | -0.6 | -1.3, 0.1 | 0.08 |
|  |  |  |  |  |
| **Wasting** | Year 5 | -1.4 | -2.0, -0.8 | <.0001 |
|  | Annual CWT | 0.7 | 0, 1.4 | 0.05 |
|  | Annual CWT x Year 5 | 0.5 | -0.1, 1.2 | 0.12 |
|  |  |  |  |  |
| **Anemia** | Year 5 | -0.2 | -0.5, 0.1 | 0.17 |
|  | Annual CWT | 0 | -0.6, 0.5 | 0.87 |
|  | Annual CWT x Year 5 | 0.3 | -0.1, 0.7 | 0.12 |
|  |  |  |  |  |
| **Increased PVD** | Year 5 | -2.1 | -3.1, -1.2 | <.0001 |
|  | Annual CWT | 0.1 | -0.5, 0.8 | 0.67 |
|  | Annual CWT x Year 5 | 0.1 | -1.1, 1.4 | 0.83 |
|  |  |  |  |  |
| **PedsQL Physical** | Year 5 | 5.4 | 3.4, 7.3 | <.0001 |
|  | Annual CWT | -0.6 | -6.6, 5.4 | 0.85 |
|  | Annual CWT x Year 5 | 1.7 | -1.1, 4.5 | 0.23 |
|  |  |  |  |  |
| **PedsQL Social** | Year 5 | 2.5 | 0.5, 4.4 | 0.01 |
|  | Annual CWT | -2.1 | -10.1, 5.8 | 0.60 |
|  | Annual CWT x Year 5 | 0.9 | -1.9, 3.6 | 0.54 |

^a^*Abbreviations*: CI, confidence interval; CWT, community-wide treatment; SBT, school-based treatment; PVD, portal vein diameter; VO_2_max, maximal oxygen uptake as estimated by fitness testing; epg, *S. mansoni* eggs per gram feces
